# Effects of Exercise Therapy on Anxiety and Depression in Patients With Coronary Heart Disease: A Meta-Analysis of a Randomized Controlled Study

**DOI:** 10.3389/fcvm.2021.730155

**Published:** 2021-10-11

**Authors:** Lina Wang, Yangli Sun, Jie Zhan, Zhiyuan Wu, Peiming Zhang, Xiaopeng Wen, Shuqi Ge, Xu Han, Liming Lu

**Affiliations:** ^1^Department of General Medicine, Zhengzhou Central Hospital Affiliated to Zhengzhou University, Zhengzhou, China; ^2^Department of Rehabilitation, Guangdong Provincial Hospital of Chinese Medicine, The Second Affiliated Hospital of Guangzhou University of Chinese Medicine, Guangzhou, China; ^3^Department of Neurological Rehabilitation, Zhengzhou Central Hospital Affiliated to Zhengzhou University, Zhengzhou, China; ^4^South China Research Center for Acupuncture and Moxibustion, Medical College of Acu-Moxi and Rehabilitation, Guangzhou University of Chinese Medicine, Guangzhou, China; ^5^Department of Urology (Henan Institute of Urology), The First Affiliated Hospital of Zhengzhou University, Zhengzhou, China; ^6^Evidence-Based Medicine and Data Science Centre, Guangzhou University of Chinese Medicine, Guangzhou, China

**Keywords:** exercise therapy, coronary heart disease, anxiety, depression, meta-analysis

## Abstract

**Objective:** The purpose of this review was to evaluate the effect of exercise therapy on anxiety and depression symptoms in patients with coronary heart disease (CHD).

**Methods:** A systematic review of papers published between January 2000 and February 2021 was conducted. MEDLINE, Embase, the Cochrane Library and Web of Science were searched. Meta-analysis was used to compare the results of the included studies. Bias risk assessment was performed using the Cochrane Collaboration bias risk tool. If half or more of the seven items in Randomized controlled trials (RCTs) were low-risk, then the RCT was considered low-risk research; otherwise, it was high-risk. Statistical analyses were performed using RevMan version 5.3 and STATA version 12.0.

**Results:** We performed a meta-analysis of 11 randomized clinical studies including 771 subjects. Eight studies (73%) were of high quality. Compared with the control group, the exercise group showed a significant improvement in anxiety [standard mean difference (SMD) = −0.61; 95% confidence interval (CI): −0.86, −0.35]. The depression level in the exercise group was also significantly reduced (SMD = −0.48; 95% CI: −0.92, −0.04). Aerobic fitness and athletic endurance also improved [mean difference (MD) = 0.77; 95% CI: 0.58, 0.95; and MD = 20.69; 95% CI: 6.91, 34.46; respectively].

**Conclusions:** This meta-analysis suggests that exercise therapy may be effective in alleviating anxiety and depression symptoms in patients with coronary heart disease. Due to methodological weaknesses, rigorous research needs to be designed to further confirm the effectiveness of exercise therapy in improving the mental health of patients with coronary heart disease.

**Systematic Review Registration:** https://inplasy.com/projects/, identifier: INPLASY202160017.

## Introduction

Partial or complete blockage of the coronary artery due to atherosclerosis, which leads to myocardial ischemia, hypoxia or necrosis, is called coronary heart disease (CHD) together with coronary spasm, and it is one of the main causes of death and life reduction worldwide (1). Depression and anxiety are recognized as social and psychological risk factors for coronary heart disease (2); ~15–20% of patients with coronary heart disease suffer from depression, resulting in increased mortality and decreased quality of life (3). The prevalence of anxiety in patients with coronary heart disease is ~15%, and a meta-analysis by Roest et al. has suggested that post-myocardial infarction anxiety increases the risk of adverse cardiac outcomes by 36% (4, 5). However, epidemiological studies have shown that <20% of patients with depression have been properly treated (6), and the rate of anxiety and depression symptoms after coronary heart disease is even lower, which will result in a higher recurrence and mortality rate of coronary heart disease, as well as high health care expenditures. The American Heart Association (AHA) published a scientific recommendation in 2008 to screen all patients with CHD for depression, and European clinical guidelines for cardiovascular diseases also recommend the treatment of depression and anxiety (7).

At present, the psychological and psychiatric intervention recommended by the guidelines seems to improve the degree of depression in patients with coronary heart disease (8–11). However, in the case of patients with coronary heart disease combined with anxiety symptoms, the effect of psychological treatment and psychotropic drugs is still unclear, indicating the need for other interventions. For patients with coronary heart disease combined with anxiety and depression, exercise may be a promising, flexible and easy-to-implement treatment option. Exercise can reduce anxiety and depression in patients without coronary heart disease as well as improve cardiac prognosis (12, 13). A meta-analysis by Kugler et al. before 2,000 suggested that exercise therapy might be beneficial to improve the mood of patients with coronary heart disease (14). However, another systematic evaluation report (15) found it impossible to definitively conclude that exercise is effective for anxiety and depression symptoms in patients with ischemic heart disease due to the general lack of data on the effect of exercise. In recent years, studies have shown that inspiratory muscle training can significantly improve anxiety symptoms after coronary artery bypass surgery (CABG) (16). In contrast, some studies have concluded that exercise-based cardiac rehabilitation has no obvious effect on improving patients' depression (17). Due to current conflicting evidence, we performed a meta-analysis to evaluate the efficacy of exercise therapy on depression and anxiety symptoms in patients with coronary heart disease.

## Methods

### Participants

All available randomized controlled trials (RCTs) met the following criteria: participants were over 18 years old and diagnosed with coronary heart disease (including acute coronary syndrome, angina pectoris, coronary artery disease confirmed by coronary angiography, percutaneous coronary intervention or coronary artery bypass grafting). An RCT was included if there were elevated levels of anxiety and depression (clinically diagnosed as anxiety and depression or scores higher than the threshold on the effective scale) as the primary or secondary outcome and there was an effective score on the anxiety and depression scale at baseline or before and after the intervention.

### Intervention

We included studies that received exercise therapy alone or as part of a comprehensive cardiac rehabilitation program that included psychological interventions or health education. Exercise could be any combination of aerobic, strength, or balance training. There were no restrictions on the length of time, frequency or method. Exercise-based cardiac rehabilitation, deep breathing exercises or inspiratory muscle training were also included.

### Comparator

If the exercise intervention was compared with standard medical care or any other intervention (such as psychological education, antidepressant drugs, or stress management), the study was included.

### Outcomes

The primary outcomes were depression and anxiety severity. Anxiety was assessed by the HADS-A, GAD 7, HAMA, and DASS-A. Depression was assessed by the HADS-D, HAMD, SDS, PHQ-9, BDI, DASS-D, and CDS, all of which are effective self-reporting tools. For example, the Hospital Anxiety and Depression Scale (HADS) consists of 14 items, seven measuring anxiety and seven measuring depression (18). According to the literature, the cutoff point for elevated anxiety and depression as measured by the HADS is eight. For example, the SDS consists of 20 items that describe depressive symptoms. Respondents describe how often they experience each symptom on a 4-level scale, from “no or few” to “most or all.” Lower scores represent a more favorable psychological state. Secondary outcomes were the 6-min walk test and peak oxygen consumption.

### Search Strategy

We searched MEDLINE, Embase, the Cochrane Library and Web of Science from 1 January 2000 to 1 February 2021. This period was chosen to select recent studies that reflect contemporary clinical cardiology practice. We established search strategies that combined medical subject headings and free-text terms that contained subjects with coronary heart disease, anxiety, depression, exercise therapy, and randomized clinical trials. The citations retrieved from these four electronic databases were further screened for potentially eligible studies. A manual search of published meta-analyses, review articles, and handbooks of meeting minutes was also performed to ensure inclusion of all relevant studies for preliminary review. The searches were limited to studies published in English. Detailed search strategies are described in Appendix 1.

### Study Selection

Two reviewers (LNW, PMZ) independently screened all titles and abstracts for potential eligible publications. Articles that passed the initial screening underwent full text review by both reviewers. Disagreement about study eligibility was resolved by discussion with the last author (JZ).

### Data Collection Process

Two reviewers (LNW, JZ) independently extracted data from each study using a predefined data extraction sheet. The information we collected included research characteristics (such as the author's name, publication year, research design, country, and sample size), population characteristics (such as average age, gender, baseline anxiety level (HADS-A, GAD7, HAMA, and DASS-A), baseline depression level (HAMD, SDS, PHQ-9, BDI, HADS-D, DASS-D, and CDS), and intervention characteristics (movement type, frequency, and duration). If studies presented insufficient data or uncertain information, we contacted the corresponding authors. Discrepancies were resolved by discussion with the third reviewer (LL).

### Risk of Bias in Individual Studies

Using the Cochrane Collaboration bias risk tool, we identified the following domains as relevant for assessing the RCTs: sequence generation, allocation concealment, blinding of participants and personnel, incomplete outcome data, selective outcome reporting, and other sources of bias. Blinding was assessed at the outcome level. Two reviewers (LNW, JZ) independently classified each domain as having a low, high or unclear risk of bias. If half or more of the seven items in an RCT are low-risk, the RCT can be considered low-risk research; otherwise, it is high-risk. Disagreement about the risk of bias was resolved by discussion with the third reviewer (LL).

### Analysis

The statistical analyses were performed using RevMan version 5.3 (Version 5.3. Copenhagen: The Nordic Cochrane Centre, The Cochrane Collaboration, 2014) and STATA version 12.0. The mean difference (MD) or standardized mean difference (SMD) with 95% CI was used to analyze continuous outcomes. The SMD statistic was selected when the outcome was assessed by the different scales. *I*^2^ statistics were calculated to assess the heterogeneity and to choose the effect model. If *I*^2^ > 50% and the *P*-value of *X*^2^ was <0.1, meaning that statistical heterogeneity existed among studies, a random-effects model was selected. Otherwise, a fixed-effects model was used. If the pooled result included clinical heterogeneity, subgroup analysis was performed to search for the source of heterogeneity. Moreover, to examine the stability of the analysis result, we also conducted a sensitivity analysis by removing one study at a time. In studies that reported multiple depression results, to prevent double counting in the meta-analysis, we randomly selected one. If a study included multiple intervention groups (with different treatments), we combined all relevant experimental interventions from the study into a single group (comparison format: all exercise groups vs. control group) and combined the mean and SD using the continuous outcome formula in the Cochrane manual (19).

## Results

### Study Selection

**Figure 1:**
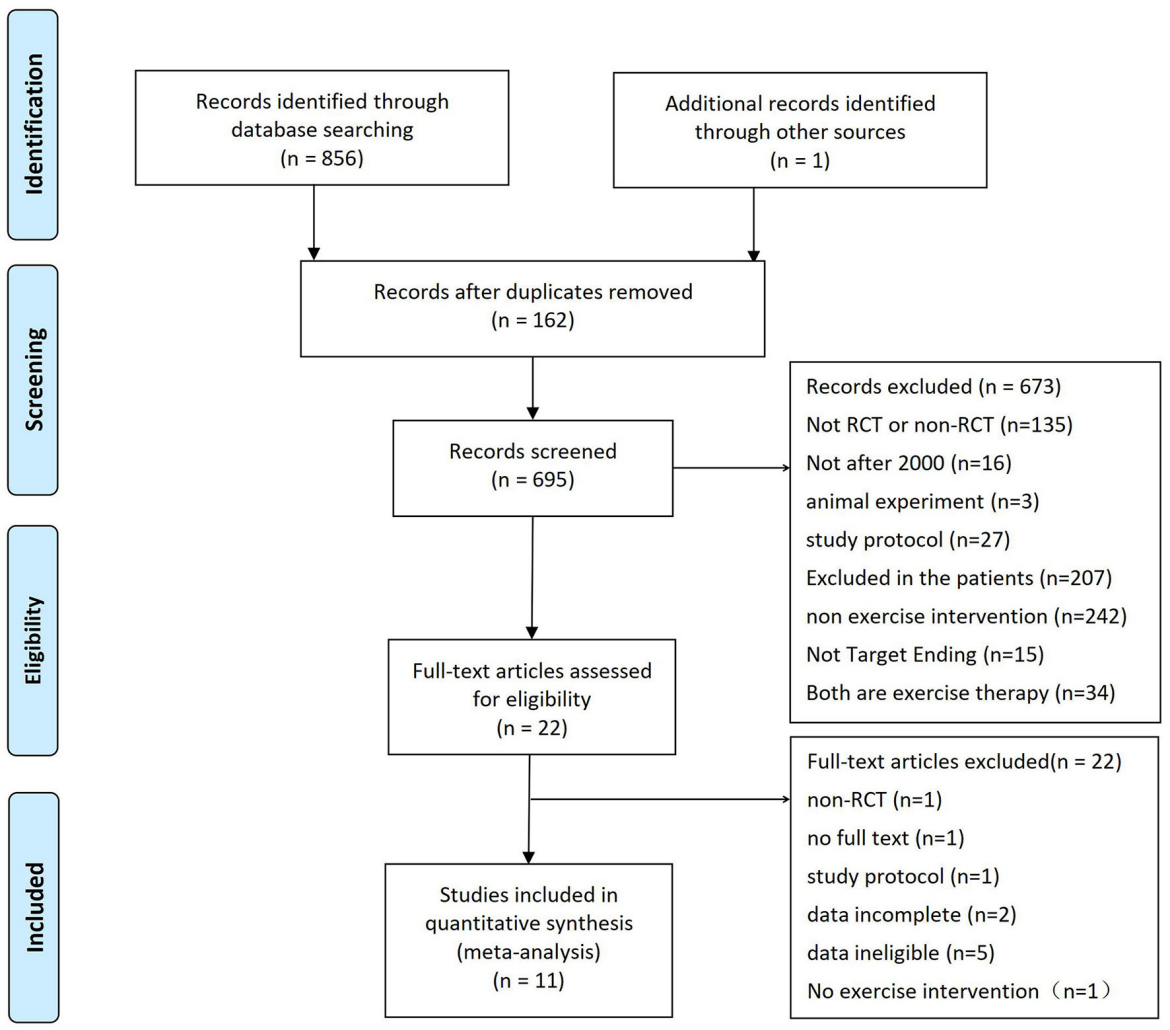
Flow of the trial selection process.

In total, 856 citations were obtained by searching 4 electronic databases along with reference lists (one citation). Overall, 158 repetitive citations were eliminated. Based on the abstracts and titles of these studies, 676 citations were excluded. Twenty-two citations passed the full-text review, and 11 were excluded because they did not meet the eligibility criteria. The remaining 11 randomized controlled trials, including 771 participants, met the eligibility criteria. In the end, 11 studies met the inclusion criteria and were included in the meta-analysis (see Figure 1).

### Design and Patient Characteristics

The baseline characteristics of the 11 eligible RCT studies are summarized in Table 1. Eleven studies were randomized controlled studies, 10 studies (91%) reported a primary outcome of depression, five studies (45%) reported a primary outcome of anxiety, the average age of patients ranged from 51.51 to 72.46 years old, and the majority of the patients were male. Treatment ranged from 10 days to 24 weeks. All the experimental treatment groups were treated with exercise therapy, while the control group was treated with non-exercise therapy.

**Table 1:** Characteristics of included studies.

**References**	**Design**	**Country**	N*	**Mean age, years**		**Mean**, ***n*** **(%)**		**Outcome**	**Anxiety assessment**	**Depression assessment**
				**Experiment**	**Control**	**Experiment**	**Control**			
Blumenthal et al. (20)	RCT	America	101	63.9		68		HAMD, peak VO2, SDNN, HRV, FMD, BRS, PF4, βTG	NR	HAMD
Seki et al. (21)	RCT	Japan	38	69.3	70.1	100	100	SF-36, STAI, SDS, HRQOL, peak VO2	NR	SDS
Ghisi et al. (22)	RCT	Canada	115	59.5		71		CADE-Q II, PHQ-9, Health behaviors	NR	PHQ-9
Blumenthal et al. (23)	RCT	America	134	63		69		GHQ, BDI, LVEF, WMA, FMD, HRV-DB, Baroreflex sensitivity	NR	BDI
Chung et al. (24)	RCT	Taiwan, China	62	72.46	70.71	82.14	58.82	BDI-II, PHQ-9	NR	PHQ-9
Fang et al. (25)	RCT	China	80	60.24	61.41	63.6	61.8	blood pressure, 6MWT, FTND, CDS, SF36	NR	CDS
Ding et al. (17)	RCT	China	80	57.15	56.57	80.77	82.14	6MWT, SF-12, PHQ-9, GAD-7, exercise adherence.	GAD7	PHQ-9
Savci et al. (16)	RCT	Turkey	43	62.82	57.48	86	86	6MWT, HADS,	HADS-A	HADS-D
Sharma et al. (26)	RCT	India	66	53.15	51.51	78.8	93.9	LVEF, CDS, HAMA, DASI, MET	HAMA	CDS
Vieira et al. (27)	RCT	Brazil	46	57.7	59	100	1000	Trail Making Test, Verbal Digit Span Test, Stroop, MacNew, DASS 21	DASS-A	DASS-D
Asbury et al. (28)	RCT	Britain	42	NR	NR	NR	NR	Physical Ability, HAQ, HADS, Health beliefs, SF-36	HADS-A	NR

### Risk of Bias Assessment

**Figure 2:**
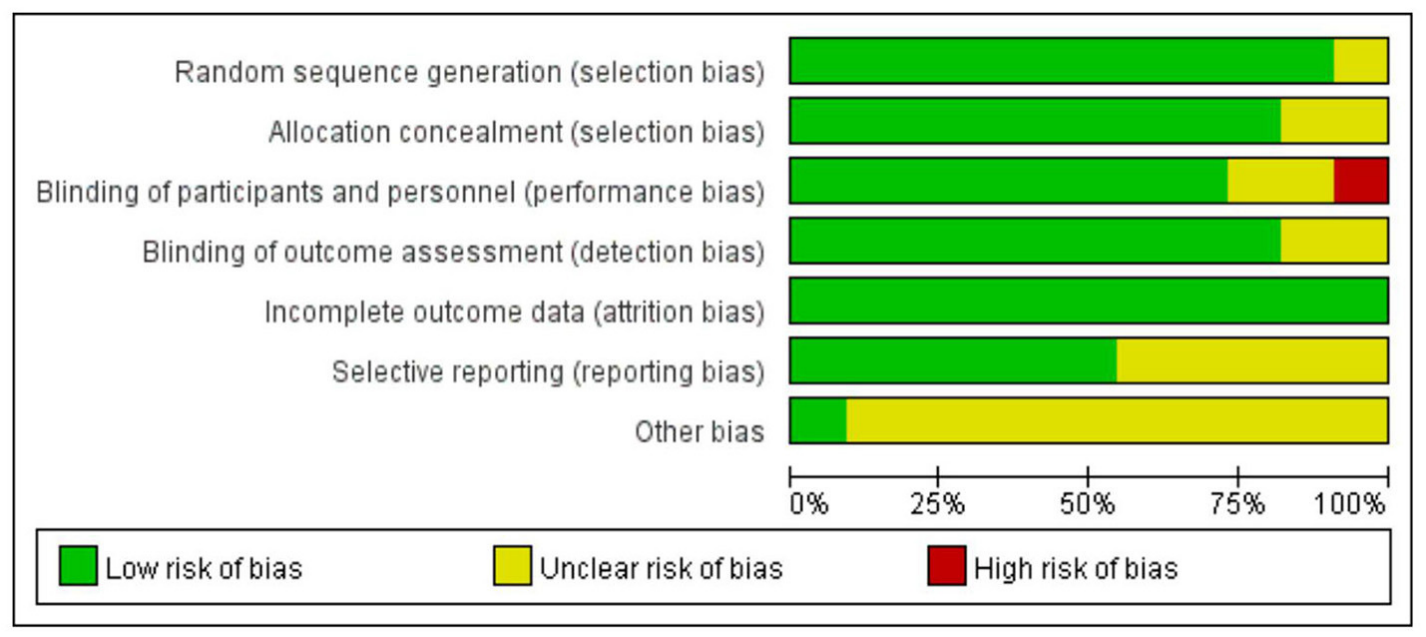
Demonstrate the risk of bias in the included studies.

**Figure 3:**
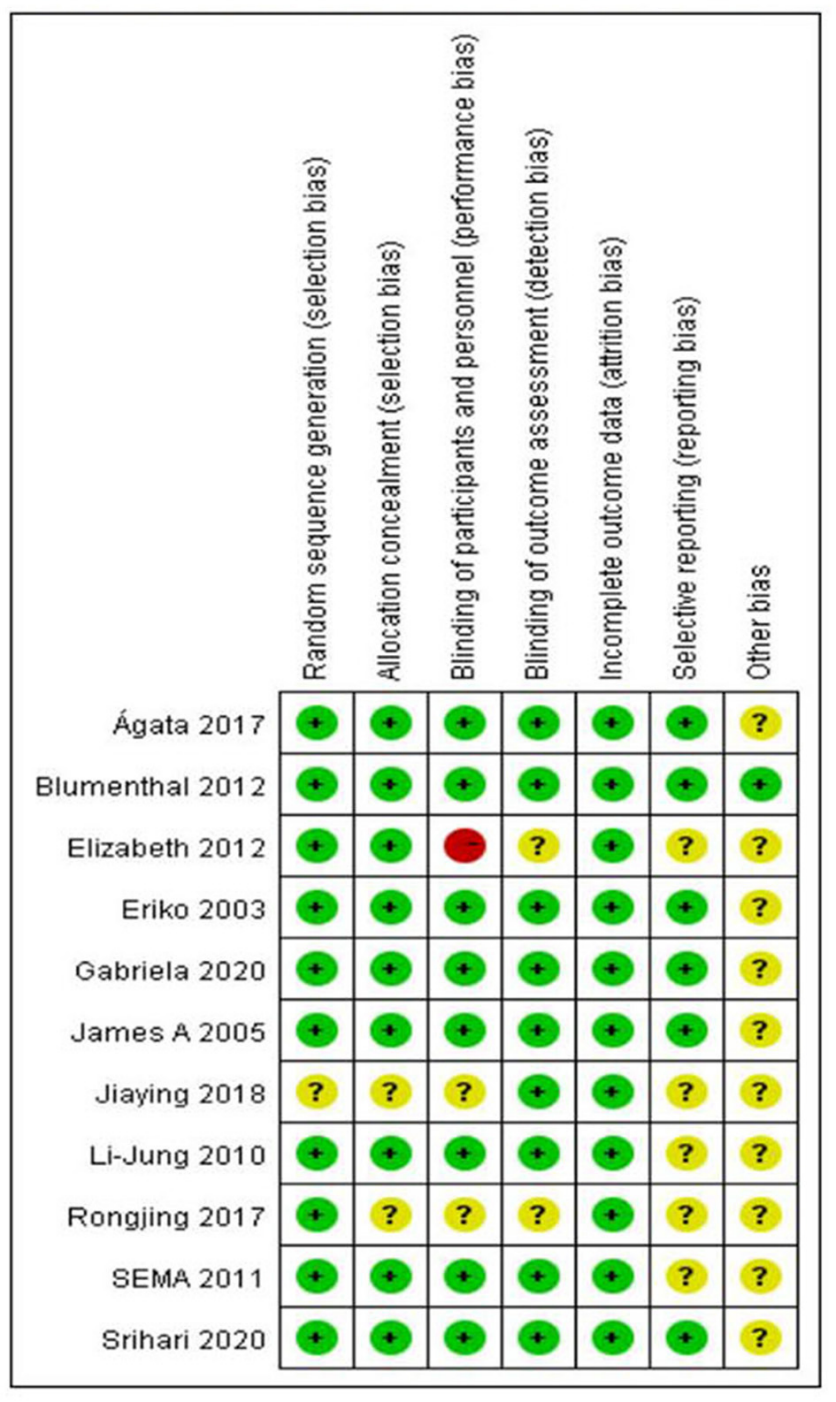
Risk of bias summary review authors' judgements about each risk of bias item for each included study.

Eight of the 11 RCTs assessed the risk of bias, where half or more of the seven items were low risk, resulting in eight (73%) of the RCTs being low risk studies. Two (18%) studies were judged to be biased due to insufficient reporting of allocation concealment, blinding of participants, blinding of outcome evaluation, and selective reporting. In addition, in one study (9%), due to issues related to obtaining fully informed consent, the local ethics committee explicitly prohibited the use of group blindness (Figures 2, 3).

### Primary Outcomes

**Figure 4:**
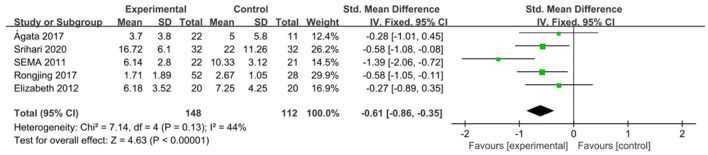
Meta-analysis: anxiety in coronary heart disease patients.

In total, 260 participants in five randomized controlled studies (16, 17, 26–28) evaluated the effect of exercise therapy on anxiety in patients with coronary heart disease (Figure 4). Using evaluation methods, including GAD7, HADS-A, HAMA, and DASS-A, due to the diversity of dimensions, we used SMD to aggregate the anxiety symptom data, and the fixed-effect model was chosen because the combined results had no obvious statistical heterogeneity. The results showed that compared with the control group, the intervention group had a significant overall effect in improving anxiety of patients with coronary heart disease.

**Figure 5:**
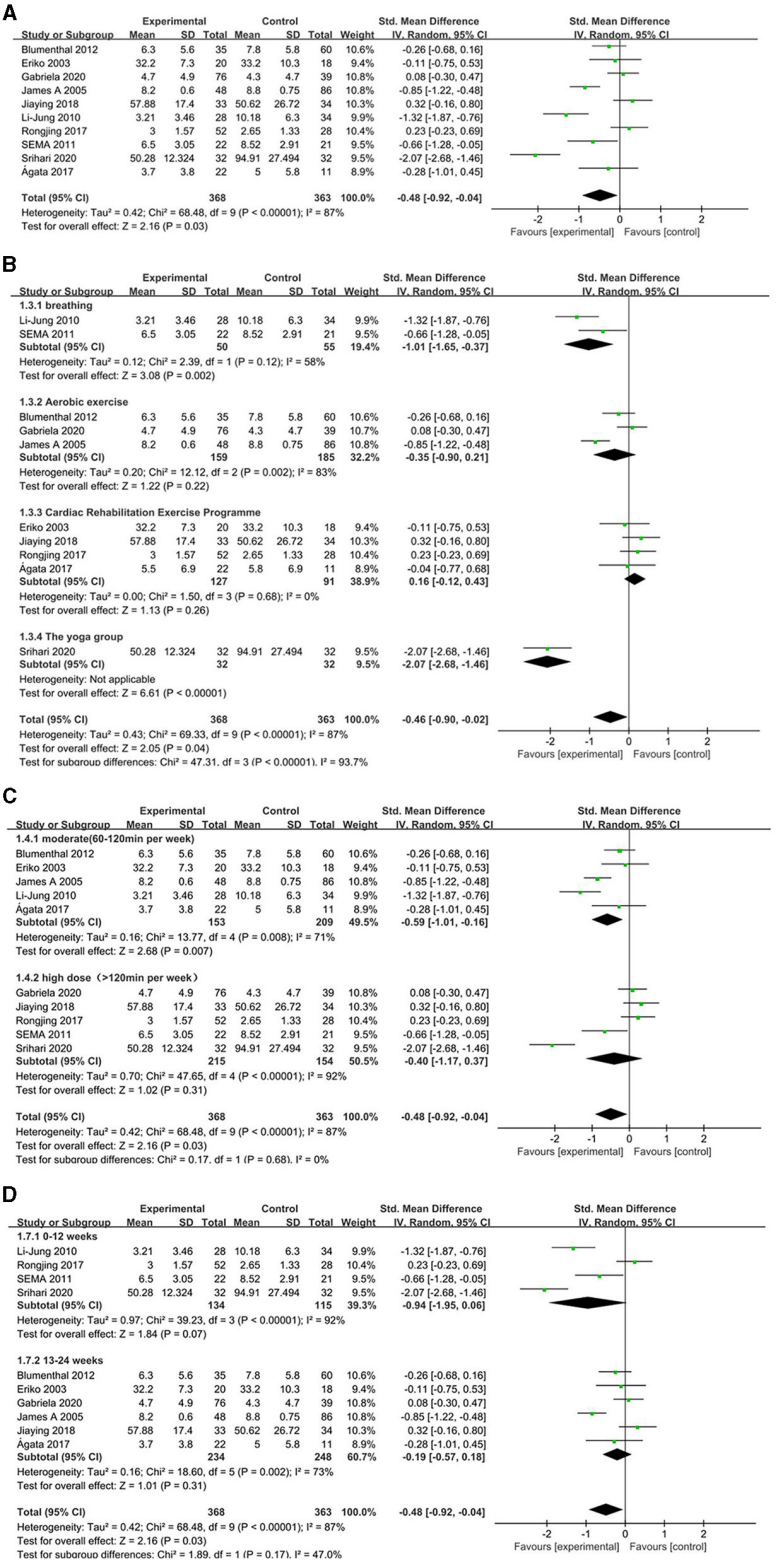
Comparison of acupuncture related therapies in terms of UPDRS-II. **(A)** The effect of exercise therapy on depression in patients with coronary heart disease. **(B)** The effect of different exercise types on depression in patients with coronary heart disease. **(C)** The effect of different exercise time on depression in patients with coronary heart disease. **(D)** According to the length of intervention, exercise therapy the impact of depression in patients with coronary heart disease.

Since one of the trials did not study the target outcome of depression and could not be included in the meta-analysis, 731 participants in 10 randomized controlled studies (16, 17, 20–27) evaluated the effect of exercise therapy on depression in patients with coronary heart disease (Figure 5). Using evaluation methods, including HAMD, SDS, PHQ-9, BDI, CDS, HADS-D, CDS, and DASS-D, the random effects model was chosen because the combined results were highly statistically heterogeneous. The comprehensive results showed that, compared with the control group, the intervention group had a significant overall effect in improving the depression of patients with coronary heart disease.

Subgroup analysis: Four exercise-focused cardiac rehabilitation studies and three studies on aerobic exercise reached no conclusions. The statistical heterogeneity of the combined results from the two studies on respiratory motion was small, and a significant difference was found between the intervention group and the control group, suggesting that the intervention group had a significant overall effect at improving the degree of depression in patients with coronary heart disease (Figure 5B). According to different intervention doses, we divided the frequency and duration of exercise therapy, and divided it into medium dose (60–120 min per week) and high dose (120 min per week). Comprehensive results of different doses show that there are differences between the exercise therapy group and the control group. There are subgroup differences, and compared with the control group, moderate-dose exercise therapy can improve depression in patients with coronary heart disease (Figure 5C). According to the length of the intervention, the treatment time is divided into 0–12 and 13–24 weeks. The results of the length of the comprehensive intervention time show that there is a difference between the exercise therapy group and the control group (Figure 5D).

**Figure 6:**
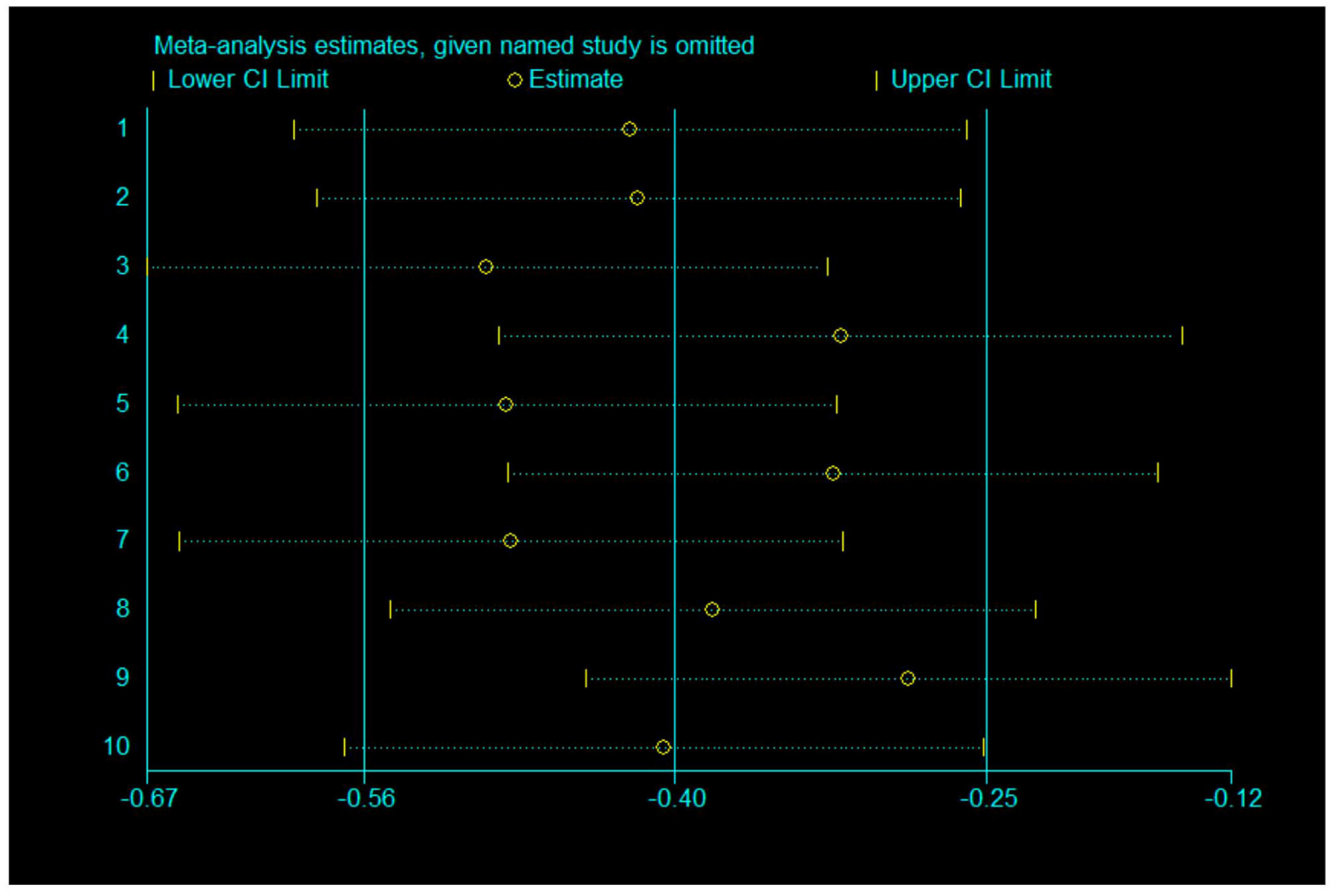
Sensitivity analysis of 10 items for depression.

**Figure 7:**
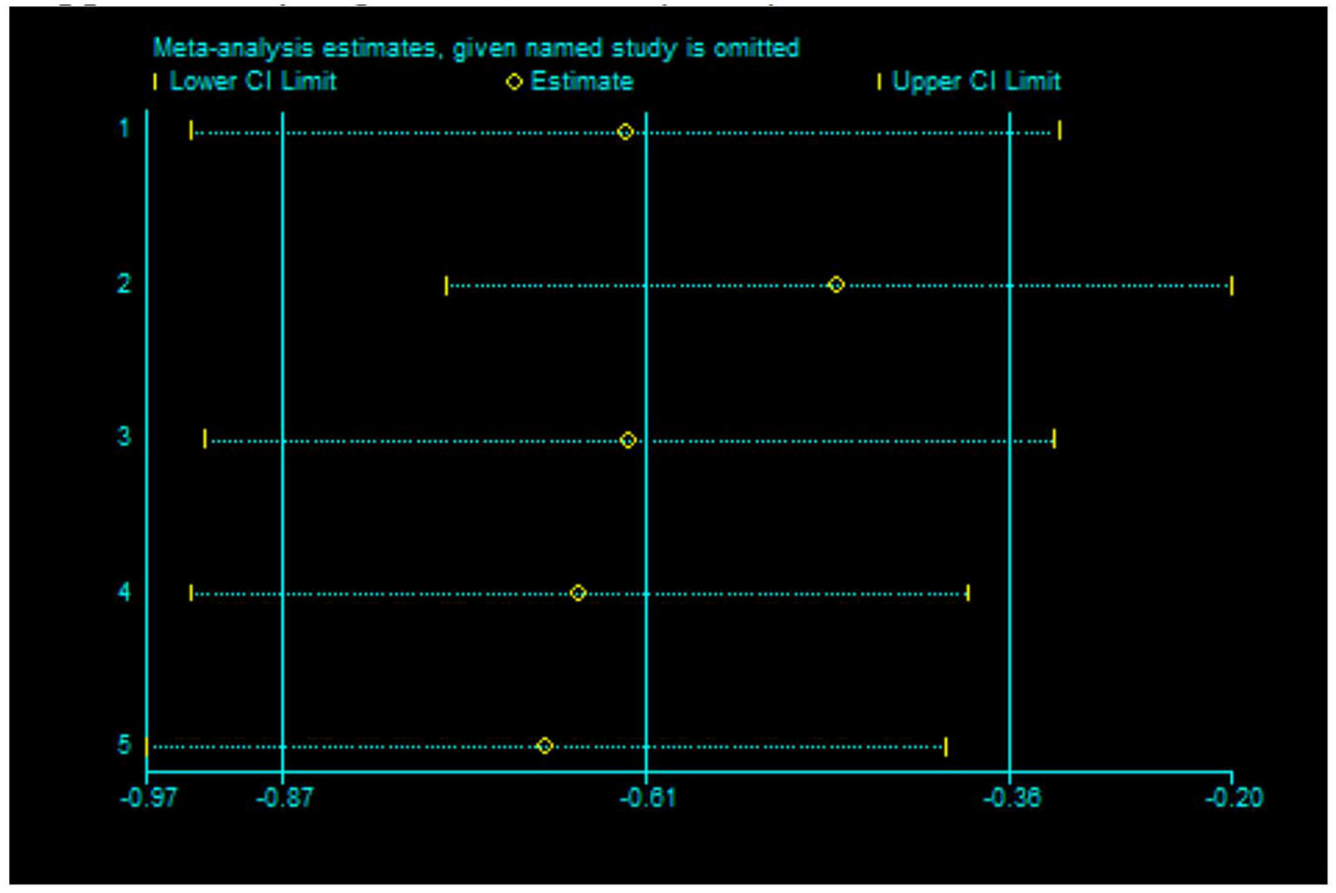
Sensitivity analysis of 5 items to the effect of anxiety.

Sensitivity analysis: A sensitivity analysis was performed by excluding one study at a time, and the results showed that after excluding one study, the combined result was stable and not affected by any single data set (Figures 6, 7).

### Secondary Outcomes

**Figure 8:**
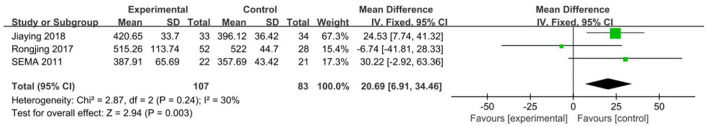
Meta-analysis: a 6-minute walk trial of exercise therapy in patients with coronary heart disease.

The cardiopulmonary function test (CPET), as the core assessment technique of cardiac rehabilitation, is a necessary condition for ensuring precise exercise prescription. In the cardiopulmonary exercise test, the peak oxygen uptake (VO2 peak) was one of the most important parameters (Figure 8). Eight of the 11 randomized controlled studies did not report the target outcome peak oxygen concentration; thus, we included 3 studies with a total of 267 participants. A meta-analysis was performed to evaluate the exercise tolerance and aerobic adaptability of exercise therapy in patients with coronary heart disease. The fixed-effects model was chosen because there was no significant statistical heterogeneity in the combined results. Compared with the control group, the intervention group had a significant difference in peak oxygen uptake.

**Figure 9:**
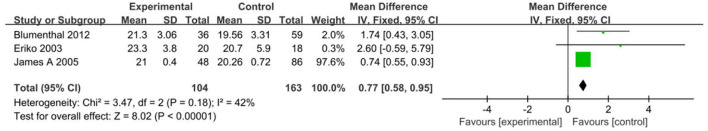
Meta-analysis: peak oxygen uptake with exercise therapy in patients with coronary heart disease.

The 6-min walk test was used to assess functional exercise ability. Of the 11 randomized controlled studies, seven studies did not report the target outcome of the 6-min walk test. One of the studies had inappropriate data. Therefore, we included three studies with a total of 190 participants for a meta-analysis to evaluate the effect of exercise therapy on coronary heart disease (Figure 9). The fixed-effects model was chosen because there was no significant statistical heterogeneity in the combined results. Compared with the control group, the intervention group had a significant difference in the 6-min walk test.

## Discussion

We performed a meta-analysis of 11 randomized clinical studies including 771 subjects. We evaluated the impact of exercise therapy on anxiety and depressive symptoms in patients with coronary heart disease, and most studies focused on patients with coronary heart disease with anxiety and/or depression at baseline. We have drawn several conclusions. First, the combined results of exercise therapy on anxiety symptom scores of patients with coronary heart disease show that the exercise group is superior to the control group in improving anxiety. Second, the combined results of exercise therapy and depression symptom scores of patients with coronary heart disease showed that the exercise group was superior to the control group in improving depression. Due to the high heterogeneity of the combined results, we conducted a subgroup analysis on different exercise methods, and the results showed that the overall effect of respiratory exercise on improving the depression degree of patients with coronary heart disease was significant, while the differences between the exercise-based cardiac rehabilitation and aerobic exercise subgroups were not statistically significant. Sensitivity analysis was performed on all main results, indicating that the results were durable and stable. Third, exercise therapy can significantly improve peak oxygen uptake in patients with coronary heart disease according to the results of the 6-min walk test. In summary, exercise therapy can improve the anxiety and depression symptoms, aerobic adaptability and functional exercise ability of patients with coronary heart disease.

Previous related studies have suggested that long-term, moderate-intensity exercise therapy can effectively alleviate depression symptoms and is widely used in the treatment of depression (29, 30). Among them, the relationship between anxiety, depression and cardiovascular disease has become an important area of research and therapeutic intervention. This relationship may affect the onset of cardiovascular disease, and the prognosis is unclear (31). The mechanism by which exercise reduces anxiety and depression symptoms may be multifactorial. Physiological effects may be an important mechanism, including (32) a reduction in inflammation, improvement in endothelial function, reduction in platelet aggregation, and sympathetic-vagal balance. Social interaction may also be an important mechanism. Most importantly, exercise therapy reduced the risk factors by improving depressive mood, which was associated with significant reductions in morbidity and mortality from coronary heart disease (33).

In this study, the therapeutic effect of exercise on depressive symptoms is affected by the way of exercise. The characteristics of different exercise interventions are shown in Table 2. A different finding is that inspiratory muscle training and home deep breathing therapy suggest improvement in depression symptoms in patients with coronary heart disease, but the antidepressant effects of exercise-based cardiac rehabilitation and aerobic exercise have not been confirmed, which is in line with previous meta-analysis The results are inconsistent (34). This inconsistency might be related to the sample size or the type of articles included. Second, in this study, the depressive symptoms were mostly mild and moderate, and failure to select RCTs with patients with high depressive symptoms might have affected the evaluation of exercise intervention, thus making it impossible to detect clinically significant changes in the entire study sample. The baseline levels of anxiety and depression in the study are shown in Tables 3, 4. Five of 11 RCTs included exercise as part of a multicomponent intervention. These results are difficult to explain because not all treatment-related changes can be attributed to exercise. Methodologically, depression and anxiety symptoms were secondary results in some studies. This issue may have affected our results, as the study may have adequately supported the primary outcome but not necessarily the secondary outcome (35). Due to the high heterogeneity of the results, we conducted a subgroup analysis based on the effect of different exercise time on depression in patients with coronary heart disease. Medium-dose exercise therapy (60–120 min per week) may be the best treatment to improve depression symptoms in patients with coronary heart disease time. For the improvement of exercise tolerance and aerobic fitness, according to previous studies, moderate intensity and frequent exercise may reduce the potential risk factors for coronary heart disease (36). The update of the American Heart Association's Scientific Statement (AHA) in 2020 also pointed out that regular exercise can bring cardiovascular benefits to most people, but it may bring harm to those who exercise intensively or are incompetent (37). The exercise intensity of the studies in this paper was mostly moderate, which is consistent with the basic recommendations of previous guidelines for exercise.

**Table 2:** Characteristics of different exercise interventions.

**References**	**Experiment**	**Control**	**Mode**	**Frequency**	**Length (weeks)**	**Intensity**
Blumenthal et al. (20)	Aerobic exercise	Sertraline	Aerobic exercise	3 times/week	16	70–85% of MHR
Blumenthal et al. (23)	Supervised aerobic exercise training	Usual care or stress management	Stretching, bicycle, walk, jogging	3 times/week	16	50–70% of HRR+ 70–85% of HRR
Savci et al. (16)	IMT	Usual care	IMT	two times per day	10 days	15 and 45% of MIP
Seki et al. (21)	Phase III CR and exercise training	Standard care	Stretching, calisthenics, walk, bicycle, jogging,	1 times/week	24	Anaerobic threshold level
Asbury et al. (28)	Home-based cardiac rehabilitation	Symptom monitoring	Brisk walking, strengthening exercises.	≥1 times/week	8	60–75% of HRR or 40–60% of HRR
Ghisi et al. (22)	Exercise-only CR	No CR	Aerobic and resistance training	1-3 times/week	24	50 and 80% of HRR
Vieira et al. (27)	Home-based phase III cardiac rehabilitation specific exercise	Routine care	Enhancing cardiorespiratory, muscular endurance, limb flexibility	3 times/week	24	Borg scale (12–13) or 65–70% of HRR
Sharma et al. (26)	Yoga	Standard care	Yoga	3 times/week	12	3.3 METs
Fang et al. (25)	Home-based cardiac telerehabilitation	Usual care	Walking, jogging	3 times/week	6	NR
Chung et al. (24)	Home-based deep breathing training	Weekly telephone support	IMT	thrice daily	4	NR
Ding et al. (17)	Home-based cardiac rehabilitation	Routine secondary prevention	Moderate levels of walking	5 days/week	12	NR

**Table 3:** Baseline of anxiety included in the study.

**References**	**Scale**	**Intervention group**	**Control group**	**MEAN**	**SD**	**MEAN**	**SD**	**When measured (weeks)**	**Diagnosis**
Ding et al. (17)	GAD7	52	28	2.43	3.18	1.76	0.77	12	Angina
Savci et al. (16)	HADS-A	22	21	7	3.94	8.62	2.91	10 days	CABG surgery
Sharma et al. (26)	HAMA	32	32	23.21	10.985	26.45	13.257	12	Stable patients with CAD
Vieira et al. (27)	DASS-A	22	11	5.9	7	6.9	7.4	24	Stable patients with CAD
Asbury et al. (28)	HADS-A	20	20	9.5	5.19	8.45	4.69	8	Refractory angina

**Table 4:** Baseline of depression included in the study.

**References**	**Scale**	**Intervention group**	**Control group**	**MEAN**	**SD**	**MEAN**	**SD**	**When measured (weeks)**	**Diagnosis**
Blumenthal et al. (20)	HAMD	35	60	13.1	5.9	13.9	6	16	Stable patients with CAD
Seki et al. (21)	SDS	20	18	34.4	8.3	32.9	8.3	24	Chronic CAD
Ghisi et al. (22)	PHQ-9	76	39	4.9	5.1	4.4	5.1	24	Stable patients with CAD
Blumenthal et al. (23)	BDI	48	86	9.8	7.7	9.4	7.8	16	Stable patients with IHD
Fang et al. (25)	CDS	33	34	70.97	21.3	66.21	18.51	6	Low risk after PCI
Chung et al. (24)	PHQ-9	28	34	5.5	4.45	9.21	5.19	4	Stable patients with CAD
Ding et al. (17)	PHQ-9	52	28	3.85	2.57	1.76	1.09	12	Angina
Savci et al. (16)	HADS-D	22	21	5.68	2.9	7.62	3.57	10 days	CABG surgery
Sharma et al. (26)	CDS	32	32	91.52	23.851	104.94	27.917	12	Stable patients with CAD
Vieira et al. (27)	DASS-D	22	11	5	4.4	4.2	3.8	24	Stable patients with CAD

Anxiety disorder is very common in patients with coronary heart disease (CHD), and there is increasing evidence that high levels of anxiety disorder are associated with poor prognosis. However, few studies have evaluated the efficacy of relieving anxiety symptoms and thus improving clinical outcomes in patients with coronary heart disease (38). As far as we know, for the first time we conducted this meta-analysis, exercise therapy significantly improved the anxiety symptom scores of patients with coronary heart disease, verifying the previous systematic reviews (6). In another meta-analysis (7), it was concluded that exercise-based cardiac rehabilitation can significantly improve anxiety, but the heterogeneity of the combined results was high, which affected the interpretation of the results. In the five anxiety studies, one on deep breathing exercise, one on inspiratory muscle training, and the other three on exercise-based cardiac rehabilitation. In terms of the quality of the included studies, 65.6% were evaluated as high-quality studies, but concealment and blindness remained, which might have weakened the robustness of the evidence to some extent.

Some limitations to this analysis should be noted. First, our analysis of anxiety and depression symptom data was limited to SMD because many different anxiety and depression scales were used in the included trials (The application of different psychological scales is shown in Table 5). Second, gender differences between populations with coronary heart disease need to be considered, as the majority of participants in this study were male, which may limit the universality of the results, especially when considering older women with coronary heart disease. Studies have shown that participation in exercise therapy can help to reduce depression and anxiety in women after myocardial infarction, while men's depression and anxiety do not change significantly. Third, due to the relatively wide range of coronary atherosclerotic heart disease, the World Health Organization divides coronary heart disease into five major categories. The research included in this META analysis may not be enough to support classification based on severity. The research has a certain pointing effect. Finally, there is a lack of tests to examine the effects of exercise alone on anxiety and depression. Large-scale, high-quality randomized controlled trials (RCTs) are needed to determine the benefits of exercise in the treatment of anxiety and depression in patients with coronary heart disease, especially in elderly patients and patients with different types of coronary heart disease.

**Table 5:** Application of different psychological scales.

**Mental scale name**	**Scoring method**	**Grading**	**Significance**
Health questionnaire depression scale (**PHQ-9**) (39)	A 4-level scoring method of 0 to 3 points is used. The standards for each level are: 0 means no; 1 means light for a few days; 2 means more than half of the time is medium; 3 almost every day; the total score is the score of 9 items Add up.	A total score of 0–4 points without depression; 5–9 points may have mild depression; 10–14 points may have moderate depression; 15–19 points may have moderate or severe depression; 20–27 points may have severe depression disease.	The 9-item patient health questionnaire depression scale is the most important depression screening scale. Including nine projects to investigate the situation in the past week
Beck depression questionnaire (**BDI**) (40)	There are 21 groups of items, each group has four sentences, and a 4-level scoring method of 0–3 points is adopted. Add the scores together to get the total score.	A total score of 0–9 is divided into no depression, 10–18 is mild depression, 19–29 is moderate depression, and 30–63 is severe depression.	It is most effective for mild to moderate depression and non-psychotic depression. Depression accompanied by physical disease or physical dysfunction also has a good effect.
Hamilton depression scale (**HAMD**) (20)	Most of the 17-item tables are used, most of which use a 5-level scoring method of 0–4 points. The criteria for each level are: (0) None; (1) Mild; (2) Moderate; (3) Severe; (4) Extremely severe.	More than 24 points are classified as severe depression, more than 17 points are classified as mild or moderate depression, and <7 points have no depressive symptoms.	HAMD is the most commonly used scale for clinical evaluation of depression. Especially suitable for depression. Scoring before and after treatment can evaluate the severity of the condition and the effect of treatment.
Self-rating depression scale (**SDS**)	Each item is divided into four grades according to the frequency of symptoms, of which 10 are positive scores and 10 are reverse scores. If it is a forward scoring question, it will be rated as 1, 2, 3, and 4 points in turn; for a reverse scoring question, it will be rated as 4, 3, 2, and 1. After the evaluation is over, add up the scores in the 20 items to get the total rough score (X), then multiply the rough score by 1.25 and take the integer part to get the standard score (Y).	According to the results of the Chinese norm, the cut-off value of the SDS standard score is 53 points, of which 53–62 are classified as mild depression, 63–72 are classified as moderate depression, and more than 73 are considered severe depression.	It is mainly suitable for adults with depressive symptoms, including outpatients and inpatients. It is only difficult to assess depression with severe delayed symptoms. At the same time, SDS is not effective for people with lower education level or lower intelligence level.
Carroll depression scale (**CDS**) (41)	Depression was measured using a 26-item CDS questionnaire, each item of which may be graded on a 7-point Likert scale ranging from strongly disagree to strongly agree.	The total CDS score is the sum of all items and ranges from 26 to 182. A cut-off score of 90 for mild depression and 100 or above has been recommended to detect individuals with more severe depression.	The 26-item Cardiac Depression Scale (CDS) is designed to assess depression in adult cardiac populations.
Depression- anxiety-stress scale (**DASS-21**)	Investigate the individual's experience of negative emotions such as depression, anxiety and stress. A 0–3 point 4-point scale is used, 0 is non-conformance, three is the most consistent or always consistent, and the sum of the seven items of each subscale multiplied by two is the score of the depression and anxiety stress scale.	Depression scale ≤9 is normal, 10–13 is mild, 14–20 is moderate, 21–27 is severe, ≥28 is very severe; anxiety scale ≤7 is normal, 8–9 It is divided into mild, 10–14 is moderate, 15–19 is severe, ≥20 is very severe.	A total of 21 items were investigated in the past week, and the individual's experience of depression, anxiety, and stress and other negative emotions were investigated.
Generalized anxiety self-rating scale (**GAD7**)	Use 0~3 points and 4 points to score, respectively 3 = almost every day; 2 = more than a week; 1 = a few days; 0 = not at all. The total score is the sum of the scores of seven items, and the total score is The range is 0–21 points.	0–4 points for normal level; 5–9 points for mild anxiety; 10–13 points for moderate anxiety; 14–18 points for moderate to severe anxiety; 19–21 points for severe anxiety.	It is used to evaluate anxiety, and regular self-evaluation can observe the trend of anxiety and the effect of treatment. Screen for mental disorders in primary care.
Hamilton Anxiety Scale (**HAMA**)	A 5-level scoring method from 0 to 4 points is used. The standards for each level are: 0 means asymptomatic; 1 means mild; 2 means moderate; 3 means severe; 4 means extremely severe. The total score is the sum of the scores of the 14 items.	If the total score exceeds 29 points, it may be severe anxiety; if it exceeds 21 points, there must be obvious anxiety; if it exceeds 14 points, there must be anxiety; if it exceeds seven points, there may be anxiety; if it is <6, the patient has no anxiety symptoms. The general demarcation score is 14 points.	It is one of the most commonly used scales in psychiatric clinics, and it is often used clinically as a basis for the diagnosis and degree of anxiety disorders.
Depression—anxiety—stress scale (**HADS**) (18)	It is divided into two subscales of anxiety and depression (Hospital Anxiety and Depression Scale Anxiety Subscale HADS-A, Hospital Anxiety and Depression Scale Depression Subscale HADS-D), which are rated by 7 items, respectively, and the scale adopts four levels Score (0–3).	0–7 points are asymptomatic; 8–10 points are suspicious; 11–21 points are definitely present; when scoring, the starting point is 8 points, that is, both suspicious and symptomatic people are positive.	It is mainly used to assist doctors in assessing the degree of anxiety and depression of hospitalized patients.
Self-rating anxiety scale (**SAS**)	Each item is divided into four grades according to the frequency of symptoms, 15 of which are positive scores and 5 are reverse scores. If it is a forward scoring question, it will be rated as 1, 2, 3, and 4 points in turn; for a reverse scoring question, it will be rated as 4, 3, 2, and 1. After the evaluation is over, add up the scores in the 20 items to get the total rough score (X), then multiply the rough score by 1.25 and take the integer part to get the standard score (Y).	According to the results of the Chinese norm, the cut-off value of SAS standard deviation is 50 points, of which 50–59 points are mild anxiety, 60–69 points are moderate anxiety, and 69 points or more are severe anxiety.	SAS is suitable for adults with anxiety symptoms. SAS can be used as a self-evaluation tool for understanding anxiety symptoms in consultation clinics.

From our aggregated results, we have obtained the potential impact on clinical practice. First of all, medical staff can recommend exercise therapy as an intervention to improve anxiety and depression in patients with coronary heart disease, which may reduce potential risk factors for coronary heart disease. Second, among all major exercise therapies, deep breathing training and inspiratory muscle training may be more suitable for improving the anxiety and depression symptoms of patients with coronary heart disease as a whole. Third, moderate-intensity exercise (60–120 min per week) may be the best exercise intensity to improve depression symptoms in patients with coronary heart disease.

## Conclusion

In summary, exercise therapy may be effective in alleviating the anxiety and depression symptoms of patients with coronary heart disease. However, these findings need to be interpreted with caution given the methodological limitations within the included studies. In order to further confirm the effectiveness of exercise therapy in improving anxiety and depression symptoms in patients with coronary heart disease, large-scale, randomized, rigorous and high-quality methodological and long-term follow-up trials are urgently needed.

## Data Availability Statement

The original contributions presented in the study are included in the article/Supplementary Material, further inquiries can be directed to the corresponding author/s.

## Author Contributions

LW wrote the manuscript. LL and YS contributed to the conception. PZ and SG search the literature. LW, JZ, and LL extracted data. ZW, XW, and XH contributed to the acquisition. All authors have read and approved the final manuscript.

## Conflict of Interest

The authors declare that the research was conducted in the absence of any commercial or financial relationships that could be construed as a potential conflict of interest.

## Publisher's Note

All claims expressed in this article are solely those of the authors and do not necessarily represent those of their affiliated organizations, or those of the publisher, the editors and the reviewers. Any product that may be evaluated in this article, or claim that may be made by its manufacturer, is not guaranteed or endorsed by the publisher.
