# Supplementary material for: Effects of Exercise Therapy on Anxiety and Depression in Patients With Coronary Heart Disease: A Meta-Analysis of a Randomized Controlled Study
Source: Front Cardiovasc Med. 2021 Oct 11;8:730155. doi: 10.3389/fcvm.2021.730155 (PMC8542712; doi:10.3389/fcvm.2021.730155)
Supplement: Supplementary file 1 [file Presentation_1.pdf]

Supplementary Search strategy for each database 2021.02.01

EMBASE/MEDLINE / Cochrane/Web of science search strategy

Pubmed:

#1 Coronary heart disease[mh] or Coronary Diseases[tiab] or Disease*, Coronary[tiab] or Coronary Heart Disease*[tiab] or Disease*, Coronary Heart[tiab] or Heart Disease*, Coronary[tiab] or Chronic Stable Angina[tiab] or Angina Pectoris, Stable[tiab] or Stable angina[tiab] or Infarction, Myocardial[tiab] or Acute Coronary Syndromes[tiab] or Coronary Intervention, Percutaneous[tiab]

#2 Mental Disorders[mh] or Mental Disorder[tiab] or Psychiatric Illness*[tiab] or Psychiatric Disease*[tiab] or Mental Illness*[tiab] or Illness, Mental[tiab] or Psychiatric Disorder*[tiab] or Diagnosis, Psychiatric[tiab] or Psychiatric Diagnosis[tiab] or Mental Disorder*, Severe[tiab] or Severe Mental Disorder*[tiab]

#3 Anxiety[mh] or Angst[tiab] or Nervousness[tiab] or Anxiousness[tiab] or Social Anxiet*[tiab] or Anxiet*, Social[tiab]

#4 Depressive Disorder[mh] or depression[mh] or Depressive Disorders[tiab] or Disorder*, Depressive[tiab] or Neurosis, Depressive[tiab] or Depressive Neurosis[tiab] or Depression*, Endogenous[tiab] or Endogenous Depression*[tiab] or Depressive Syndrome*[tiab] or Syndrome*, Depressive[tiab] or Depression*, Neurotic[tiab] or Neurotic Depression*[tiab] or Melancholia*[tiab] or Unipolar Depression*[tiab] or Depression*, Unipolar[tiab] or Depressive Symptom*[tiab] or Symptom*, Depressive[tiab] or Emotional Depression*[tiab] or Depression*, Emotional[tiab]

#5 #2 OR #3 OR #4

#6 #1 AND #5

#7 Exercise Therapy[mh] or Exercise[mh] or walking[mh] or sports[mh] or dancing[mh] or Therap*, Exercise[tiab] or Rehabilitation Exercise*[tiab] or Physical Activit*[tiab] or Physical Exercise*[tiab] or Acute Exercise*[tiab] or Exercise*, Isometric[tiab] or Isometric Exercise*[tiab] or Aerobic Exercise*[tiab] or Exercise Training*[tiab]

#8 Interval training[tiab] or sport*[tiab] or movement therap*[tiab] or stretching[tiab] or dancing[tiab] or "Tai Ji"[tiab] or "Tai Chi"[tiab] or "Tai-Ji"[tiab] or "Tai-Chi"[tiab] or walking[tiab] or yoga[tiab] or yogic[tiab] or asana[tiab] or pranayama[tiab] or Cycle[tiab] or walk[tiab] or Treadmill[tiab]

#9 #7 OR #8

#10 Randomized controlled trial[pt] OR controlled clinical trial[pt] OR randomized[tiab] OR placebo[tiab] OR randomly[tiab] OR trial[tiab] OR groups[tiab]

#11 #6 AND #9 AND #10 （限定 2000 年之后，177 篇）

EMBASE:

#1 'Coronary heart disease'/exp OR 'Coronary Diseases':ab,ti OR 'Disease?, Coronary':ab,ti OR 'Coronary Heart Disease?':ab,ti OR 'Disease?, Coronary Heart':ab,ti OR 'Heart Disease?, Coronary':ab,ti OR 'Chronic Stable Angina':ab,ti OR 'Angina Pectoris, Stable':ab,ti OR 'Stable angina':ab,ti OR 'Infarction, Myocardial':ab,ti OR 'Acute Coronary Syndromes':ab,ti OR 'Coronary Intervention,

Percutaneous':ab,ti
#2 'Mental Disorders'/exp OR 'Anxiety'/exp OR 'depression'/exp OR 'Depressive Disorder'/exp
#3 'Mental Disorder':ab,ti OR 'Psychiatric Illness':ab,ti OR 'Psychiatric Disease?':ab,ti OR 'Mental Illness':ab,ti OR 'Illness, Mental':ab,ti OR 'Psychiatric Disorder?':ab,ti OR 'Diagnosis, Psychiatric':ab,ti OR 'Psychiatric Diagnosis':ab,ti OR 'Mental Disorder?, Severe':ab,ti OR 'Severe Mental Disorder?':ab,ti OR 'Angst':ab,ti OR 'Nervousness':ab,ti OR 'Anxiousness':ab,ti OR 'Social Anxiet*':ab,ti OR 'Anxiet*, Social':ab,ti OR 'Depressive Disorders':ab,ti OR 'Disorder?, Depressive':ab,ti OR 'Neurosis, Depressive':ab,ti OR 'Depressive Neurosis':ab,ti OR 'Depression?, Endogenous':ab,ti OR 'Endogenous Depression?':ab,ti OR 'Depressive Syndrome':ab,ti OR 'Depression, Neurotic':ab,ti OR 'Melancholia':ab,ti OR 'Unipolar Depression':ab,ti OR 'Depressive Symptom':ab,ti OR 'Emotional Depression':ab,ti
#4 #2 OR #3
#5 #1 AND #4
#6 'Exercise Therapy'/exp OR 'Exercise'/exp OR 'walking'/exp OR 'sports'/exp OR 'dancing'/exp OR 'Therap*, Exercise':ab,ti OR 'Rehabilitation Exercise?':ab,ti OR 'Physical activity':ab,ti OR 'Physical Exercise?':ab,ti OR 'Acute Exercise?':ab,ti OR 'Exercise?, Isometric':ab,ti OR 'Isometric Exercise?':ab,ti OR 'Aerobic Exercise?':ab,ti OR 'Exercise Training':ab,ti
#7 'Interval training':ab,ti OR 'sport':ab,ti OR 'movement therap*':ab,ti OR 'stretching':ab,ti OR 'dancing':ab,ti OR 'Tai Ji':ab,ti OR 'Tai Chi':ab,ti OR 'Tai-Ji':ab,ti OR 'walking':ab,ti OR 'yoga':ab,ti OR 'yogic':ab,ti OR 'asana':ab,ti OR 'pranayama':ab,ti OR 'Cycle':ab,ti OR 'walk':ab,ti OR 'Treadmill':ab,ti
#8 #6 OR #7
#9 'randomized controlled trial'/exp OR 'Clinical Study'/exp OR 'Clinical trial'/exp OR 'controlled clinical trial'/exp OR 'Case Control Study'/exp OR 'comparative study'/exp OR 'Equivalence Trial'/exp OR 'Pragmatic clinical trial':ab,ti OR 'randomized controlled trial (topic)'/exp OR 'clinical trials (topic)'/exp OR 'controlled clinical trial (topic)'/exp OR 'Equivalence Trial (topic)'/exp OR 'Clinical Trial$, Randomized':ab,ti OR 'Trial$, Randomized Clinical':ab,ti OR 'Comparative Stud*':ab,ti OR 'Practical Clinical Trial$':ab,ti OR 'Clinical Trial$, Practical':ab,ti OR 'Trial$, Practical Clinical':ab,ti OR 'Pragmatic Trial$':ab,ti OR 'Trial$, Pragmatic':ab,ti OR 'Pragmatic Clinical Trial$':ab,ti OR 'Random allocation':ab,ti OR 'Allocation,Random':ab,ti OR 'Randomization'/exp OR 'Random*':ab,ti OR 'Placebo':ab,ti OR 'trial$':ab,ti OR 'Case-control':ab,ti OR 'Case Control':ab,ti OR ((Case-Comparison OR Case-Compeer OR Case-Referrent OR 'Case Referrent' OR Case-Base OR 'Case Base') Stud*):ab,ti OR (Stud*, (Case-Comparison OR Case-Compeer OR Case-Referrent OR 'Case Referrent' OR Case-Base OR 'Case Base')):ab,ti OR 'comparative study':ab,ti
#10 #5 AND #8 AND #9 AND ([article]/lim OR [article in press]/lim) AND [humans]/lim AND [clinical study]/lim AND ([controlled clinical trial]/lim OR [randomized controlled trial]/lim) （169 篇）

Cochrane Library search strategy:
#1 MeSH descriptor: [Coronary heart disease] explode all trees
#2 ("Coronary Diseases" OR "Disease, Coronary" OR "Coronary Heart Diseases" OR "Diseases, Coronary Heart" OR "Heart Disease, Coronary" OR "Chronic Stable Angina" OR "Angina Pectoris, Stable" OR "Stable angina" OR "Infarction, Myocardial" OR "Acute Coronary Syndromes" OR "Coronary Intervention, Percutaneous"):ti,ab,kw
#3 #1 or #2
#4 MeSH descriptor: [Mental Disorders] explode all trees
#5 MeSH descriptor: [Anxiety] explode all trees
#6 MeSH descriptor: [Depressive Disorder] explode all trees
#7 MeSH descriptor: [depression] explode all trees
#8 ("Mental Disorder" OR "Psychiatric Illness" OR "Psychiatric Disease" OR "Mental Illness" OR "Illness, Mental" OR "Psychiatric Disorder" OR "Diagnosis, Psychiatric" OR "Mental Disorder, Severe" OR "Psychiatric Diagnosis" OR "Severe Mental Disorder" OR "Angst" OR "Nervousness" OR "Anxiousness" OR "Social anxiet" OR "Disorder, Depressive" OR "Neurosis, Depressive" OR "Depressive Neurosis" OR "Depression, Endogenous" OR "Endogenous Depression" OR "Depressive Syndrome" OR "Neurotic Depression" OR "Melancholia" OR "Unipolar Depression" OR "Depressive Symptom" OR "Depression, Emotional"):ti,ab,kw
#9 #4 OR #5 OR #6 OR #7 OR #8
#10 MeSH descriptor: [Exercise] explode all trees
#11 MeSH descriptor: [Exercise Therapy] explode all trees
#12 MeSH descriptor: [walking] explode all trees
#13 MeSH descriptor: [sports] explode all trees
#14 MeSH descriptor: [dancing] explode all trees
#15 ("Therapy, Exercise" OR "Rehabilitation Exercise" OR "Physical Activity" OR "Physical Exercise" OR "Acute Exercise" OR "Exercise, Isometric" OR "Isometric Exercise" OR "Aerobic Exercise" OR "Exercise Training" OR "Interval training" OR "sport" OR "movement therapy" OR "stretching" OR "dancing" OR "Tai Ji" OR "Tai Chi" OR "Tai Chi" OR "Tai-Chi" OR "walking" OR "yoga" OR "yogic" OR "asana" OR "pranayama" OR "Cycle" OR "walk" OR "Treadmill"):ti,ab,kw
#16 #10 OR #11 OR #12 OR #13 OR #14 OR #15
#17 MeSH descriptor: [Randomized Controlled Trial] explode all trees
#18 MeSH descriptor: [Clinical Study] explode all trees
#19 MeSH descriptor: [Clinical Trial] explode all trees
#20 MeSH descriptor: [Controlled Clinical Trial] explode all trees
#21 MeSH descriptor: [Comparative Study] explode all trees
#22 MeSH descriptor: [Equivalence Trial] explode all trees
#23 MeSH descriptor: [Clinical Studies as Topic] explode all trees
#24 MeSH descriptor: [Clinical Trials as Topic] explode all trees
#25 MeSH descriptor: [Controlled Clinical Trials as Topic] explode all trees
#26 MeSH descriptor: [Equivalence Trials as Topic] explode all trees
#27 MeSH descriptor: [Pragmatic Clinical Trials as Topic] explode all trees

#28 MeSH descriptor: [Case-Control Studies] explode all trees
#29 MeSH descriptor: [Random Allocation] explode all trees
#30 ("randomized controlled trial" OR "Clinical Study" OR "Clinical trial" OR "controlled clinical trial" OR "comparative study" OR "Equivalence Trial" OR "Pragmatic clinical trial" OR "Clinical Trials, Randomized" OR "Trials, Randomized Clinical" OR "Controlled Clinical Trials, Randomized" OR "Clinical Trial as Topic" OR "Comparative Studies" OR "Naturalistic Randomized Clinical Trial" OR "Practical Clinical Trials" OR "Clinical Trials, Practical" OR "Pragmatic Trials" OR "Trials, Pragmatic" OR "Pragmatic Clinical Trials" OR "Clinical Trials, Pragmatic" OR "Trials, Pragmatic Clinical" OR "Clinical study" OR "Clinical trial" OR "Pragmatic clinical trial" OR "Allocation, Random" OR "Randomization" OR "Randomized" OR "Placebo" OR "randomly" OR "trial" OR "Case-control" OR "Case-Control Study" OR "Studies, Case-Control" OR "Study, Case-Control" OR "Case-Comparison Studies" OR "Case-Comparison Study" OR "Case-Compeer Studies" OR "Case-Base Studies" OR "Case Base Studies" OR "Studies, Case-Base" OR "Case Control Studies" OR "Case Control Study" OR "Studies, Case Control" OR "Study, Case Control" OR "comparative study" OR "risk factors" OR "case control"):ti,ab,kw
#31 #17 OR #18 OR #19 OR #20 OR #21 OR #22 OR #23 OR #24 OR #25 OR #26 OR #27 OR #28 OR #29 OR #30
#32 MeSH descriptor: [Animal Experimentation] explode all trees
#33 (animal experiment or animal):ti,ab,kw
#34 #32 OR #33
#35 #31 NOT #34
#36 #3 and #9 and #16 and #35（47 篇）

Web of science:

TS=(Coronary heart disease OR Coronary Diseases OR Stable angina OR Infarction, Myocardial OR Acute Coronary Syndromes OR Coronary Intervention, Percutaneous) AND TS=(Anxiety OR Anxiety disorders OR Depressive Disorder OR depression) AND TS=(Exercise Therapy OR Exercise OR walking OR sports OR Rehabilitation Exercise OR Physical activity OR Physical Exercise OR Aerobic Exercise OR Exercise Training OR movement therapy OR stretching OR Tai Ji OR yoga OR pranayama OR Cycle OR Treadmill) AND TS=(Randomized controlled trial OR RCT) 463 篇
